# A novel WGF-LN based edge driven intelligence for wearable devices in human activity recognition

**DOI:** 10.1038/s41598-023-44213-4

**Published:** 2023-10-19

**Authors:** S. R. Menaka, M. Prakash, S. Neelakandan, Arun Radhakrishnan

**Affiliations:** 1 Department of Information Technology, KSR College of Engineering, Tiruchengode, India; 2 School of Computer Science and Engineering, Vellore Institute of Technology, Vellore, India; 3 Department of Computer Science and Engineering, R.M.K. Engineering College, Kavaraipettai, Tamil Nadu India; 4 Faculty of Electrical and Computer Engineering, Jimma Institute of Technology, Jimma University, Jimma, Ethiopia

**Keywords:** Health care, Health occupations

## Abstract

Human activity recognition (HAR) is one of the key applications of health monitoring that requires continuous use of wearable devices to track daily activities. The most efficient supervised machine learning (ML)-based approaches for predicting human activity are based on a continuous stream of sensor data. Sensor data analysis for human activity recognition using conventional algorithms and deep learning (DL) models shows promising results, but evaluating their ambiguity in decision-making is still challenging. In order to solve these issues, the paper proposes a novel Wasserstein gradient flow legonet WGF-LN-based human activity recognition system. At first, the input data is pre-processed. From the pre-processed data, the features are extracted using Haar Wavelet mother- Symlet wavelet coefficient scattering feature extraction (HS-WSFE). After that, the interest features are selected from the extracted features using (Binomial Distribution integrated-Golden Eagle Optimization) BD-GEO. The important features are then post-processed using the scatter plot matrix method. Obtained post-processing features are finally given into the WGF-LN for classifying human activities. From these experiments, the results can be obtained and showed the efficacy of the proposed model.

## Introduction

"Activity" refers to changes in the position of the body or limbs over time and against gravity. Both methods are effective. Human Activity Recognition (HAR) has grown in popularity as a study issue over the last two decades^1^. "Activity recognition" relates to how people use their senses to identify and classify various types of activities^2^. It enables computers to aid people in medical, rehabilitative, creative, sports, and other domains^3,4^. An successful HAR system can identify patients in medical emergencies, provide them with the care they require, and keep an eye on them to promote good behaviour^5^.

HAR prioritises raw sensor data. The algorithm recognises human behaviours automatically^6^. On-body or ambient sensors can be used to capture the deceased's movement patterns and past activity. These specifics will be revealed later. Sensor-based gadgets are used to track human behaviour. partly because of privacy concerns about cameras in intimate areas Sensors benefit from its pervasiveness. Because to smart technology and the Internet of Things (IoT), previously stationary items such as cars and walls, as well as previously movable objects such as phones and watches, can now contain sensors. Sensors record information on human movement invisibly everywhere we go. The data from each sensor is forwarded to the recognition system. Signal segmentation, noise reduction, and resampling are all part of sensor signal pre-processing. These stages are required for the succeeding processes, such as feature extraction, to succeed^7^.

ML algorithms commonly utilise heuristic manual feature extraction to identify human behaviour. Humans are unable to reach their full potential due of their specialised knowledge^8^. As a solution, researchers created a DL system that can capture sensor features while being trained. This sample includes both granular temporal data and high-level abstract sequences. CNN, LSTM, DBN, RNN, and its variants are among of the approaches developed in recent years for this purpose. Each strategy has benefits and drawbacks. DL algorithms forecast quickly, extract characteristics from raw sensor data, and learn^9^. That is one reason to buy it. Despite advances in accuracy brought about by current neural networks, traditional approaches continue to struggle with correctly predicting actions due to outmoded feature extortion tactics. This makes problem solving more difficult. The study proposes a novel solution to this challenge. Because it is built on WGF-LN, this programme can recognise human faces.

Traditional machine learning (ML) methods, including Support Vector Machines (SVM), Decision Trees, Random Forests, k-Nearest Neighbors (k-NN), and others, are frequently used in human activity detection algorithms. These methods involve developing features, such as mean, standard deviation, frequency domain characteristics, etc., that reflect various properties of sensor data^10^. The fundamental constraint of these approaches is that manually constructing valuable features can be time-consuming and may only capture some of the intricate patterns in the data. In particular, recurrent neural networks (RNNs) and convolutional neural networks (CNNs) have demonstrated outstanding performance in various pattern detection tasks, including identifying human activities. Without manually creating features, these models can learn hierarchical features from unprocessed sensor data^11^. RNNs are more suitable for sequential data, while CNNs are better suited for spatial properties in image-like data. RNN designs like Long Short Term Memory (LSTM) and Gated Recurrent Units (GRUs) are frequently employed in applications requiring activity recognition.

The proposed "A novel WGF-LN Based Edge Driven Intelligence for Wearable Devices in Human Activity Recognition" is unique in that it incorporates WGF-LN (Wavelet-based Graph Filter with Laplacian Normalization) technology into the realm of wearable devices and human activity recognition. Instead of depending entirely on centralized processing, this cutting-edge solution leverages the potential of edge computing, allowing wearable devices to do real-time data analysis directly on the device itself. The system delivers greater feature extraction and noise reduction using WGF-LN, a sophisticated fusion of wavelet transformation, and graph filtering. This results in improved accuracy and efficiency in recognizing human actions. This approach not only overcomes the constraints of traditional systems that mainly rely on cloud-based processing but also improves user privacy by reducing the need to transfer sensitive data. As a result, our unique edge-driven intelligence framework represents a substantial development in wearable technology, paving the door for more responsive and dependable human activity recognition systems.

### Problem definition

Existing methodologies have many ideas to solve the issues presented in the human activity recognition system. But still, more inducements are needed to improve due to the enlisted problems below,

- In previous DL techniques, there is no data available to train the system, and also Due to the unreliability of the patient's location information, the DL model suffers from imbalance problems and inaccurate classification.
- While adopting DL, in finding local solutions at the edge layer, there is a requirement for continuous data adjustment, and this problem has not yet been concentrated and solved.
- As edge intelligence is intended to have low latency but due to communication overhead and intermediate data transmission, it gets higher. This is to be reduced further.

This study proposes a WGF-LN-based edge-driven intelligence to recognise human activity on a wearable device.

This section contains a summary of the remaining text in the article. "Literature survey" of this work discusses the methods for spotting human activities. The proposed method for identifying human behaviors is covered in "Proposed methodology for human activity recognition system". In "Result and discussion", we will go over our performance-based methodology. The study's conclusion identifies next steps.

## Literature survey

In a 2020 study, Zhou^12^ and colleagues demonstrated how human activity recognition with DL enhancements can aid IoT medical devices. A classification system based on long short-term memory (LSTM) was created to recognise granular patterns in sequential motion data. We accomplished this through the analysis of sequential motion data. It was possible to merge on-body, environmental, and individual profiles by fusing data from multiple sensors. The created method was shown to be beneficial and effective in experiments and assessments utilising real-world data, however it was unable to perform high-level recognition tasks for complicated activity recognition.

Maswadi^13^ recommend Decision Tree and Naive Bayes classifiers for classifying human activities. Before being used for training and classification, raw accelerometer data needed to be noise-freeed. To process raw data, a sliding window algorithm, a Gaussian filter, and a linear filter were used. Then, using Naive Bayes and Decision Tree algorithms, human activities such as standing up, walking, sitting down, and standing up were categorized. The Gaussian filter along with the NB and DT classifiers produced the best results. Because these techniques were unable to comprehend the relationships between the features, performance could not be improved.

The overall survey of wearable devices in human activity recognition with their methods are accuracy are presented in the Table 1.

**Table 1:** Survey of wearable devices in human activity recognition.

Author	Title	Method	Accuracy	Year
Shang et al.^34^	LSTMCNN detects The use of human activity in WiFi CSI data	DL framework, LSTM-CNN	Multi-activity classification has an average accuracy of 94.14%	2021
Slim et al.^35^	An evolutionary method for optimising IoT DL parameters	optimization of DL using Genetic Algorithm	It achieved 94.5% accuracy	2021
Ihianle et al.^36^	DL can recognise human activities by using data from several sorts of sensors	CNN + BLSTM	97.8%	2020
Ghate^37^	hybrid DL algorithms for smartphone-based human activity recognition	Hybrid DL approaches	97.77%	2021
Nafea et al.^38^	Sensor-based, spatial–temporal DL is used to recognise human activity	Spatio-Temporal DL	98.53%	2021
Sanguannarm et al.^39^	LSTM and accelerometer data enable human activity recognition experiments	LSTM	N/A	2021
Mekruksavanich S^40^	Using data from smartwatch sensors, a CNN-LSTM network can recognise daily activities	CNN-LSTM	achieved 96.87% accuracy	2021

A web-based, open-access IRS database is kept up by the Communications, Sensing, and Imaging group at the University of Glasgow in the United Kingdom^14^. This is the first study to use sophisticated algorithms like Bagging and Decision Tree with open IRS data. The IRS was experiencing signal transmission and reception issues.

A unique adaptive design that employs an output block predictor was provided to select which components of the baseline architecture should be used during the inference stage^15^. The developed model proceeded with the steps of pre-processing, segmentation, and down sampling. Following these operations, Convolutional Neural Network (CNN) was deployed to classify the activity. According to experimental findings, adaptive designs are more energy-efficient and provide performance that is comparable to or better than baseline architectures. Traditional adaptive architectures suffer from performance loss. But, the model is easily suspected of an overfitting problem since using a large number of data.

The smart phone gathered sensory data, processed it with high-efficiency features, and then used multiple three-axis accelerometers to gather information about the user's physical behavior. CNN, a traditional neural network technique, is still useful in recognising human behavior^16^. This was discovered thanks to CNN's classification and identification abilities.

A federated learning^17^ system that is tailored to identify human behavior. FedHAR uses FL to create a scalable, privacy-aware global activity model. FedHAR used active learning and label propagation to annotate unlabeled sensor data. This was accomplished through the use of active learning and label propagation. According to the study, the recognition rates for solutions under active learning and label propagation are the same. To employ the label propagation model, the feature vectors must be graphed.

By merging fuzzy aggregation and fuzzy temporal windows, fuzzy cloud-fog was produced^18^. Our cutting-edge fuzzy cloud-fog method detects activity in smart homes. Fog nodes were used in this discussion to refer to smart home hardware. These fog nodes recognised activities and delivered ambient-assisted living solutions via fuzzy cloud-fog computing. Finally, a comparison of the fuzzy approach to cutting-edge techniques demonstrated its advantages. The vagueness and fuzzy nature of smart device connections hampered data processing.

It was suggested that understanding daily life be broken down into countable and uncountable actions^19^. Using the global, local, and their combinations features, activities can be classified as countable or non-countable. Following feature extraction, we selected the most useful and relevant characteristics for HAR. In comparison The strategy improved model precision over previous methods. The system required costly upkeep.

A novel approach to smart healthcare that makes more use of AI and current technology. Deep reinforcement learning reduced reaction time, latency, and congestion (DRL)^20^. To protect the privacy of medical data, the proposed methodology featured a reinforcement learning (RL) offloading mechanism. Experiments proved how rapidly the system responded. Compression still resulted in significant data loss.

Using PPG^21^ and accelerometer data, human activity detection in embedded devices was demonstrated. To account for motion artefacts in PPG and reliably detect human activity, were integrated by a Recurrent Neural Network (RNN). According to research, this process may work when resources are scarce. More RAM was required for the data.

CNN-GRU^22^ is a deep neural network model for human action recognition. Convolutional and gated recurrent units are combined in this model. During preprocessing, the sliding window method was used for data transformation. According to the findings, hybrid DL models can automatically extract geographical and temporal data to recognise complex human behaviours. Due to the neural network's limited learning capacity, the created system was incorrect.

Recognition of Activity Utilizing MCBAR (Multimodal Channel State Data) Multimodal Generative Adversarial Networks (GAN) and the marginal loss generator are used by MCBAR to increase system robustness^23^. The system is more adept at controlling environmental dynamics than the human activity identification methods now in use, according to experiments. Training GAN was challenging.

A convolutional layer, long-term and short-term storage, and a neural network (LSTM). Convolution layers were added after the two-layer LSTM model had been fed with information from mobile sensors^24^. After convolution, the fully connected layer was used with a global average pooling layer to lower the model's parameters. According to certain studies, the developed model was more durable and capable of identifying actions than others. The system was erroneous because the authors ignored challenging aspects.

A genetic algorithm can recognise human actions using fog computing frameworks. DL was used to determine the updated frame's action^25^. The action from the first frame continued into the second, and nothing else changed. An acceleration-based genetic algorithm (GA) was employed on freshly created subblocks. Experiments were used to illustrate the system's increased effectiveness. These systems can be time- and money-consuming to setup and maintain.

An improved Bayesian Convolution Network^26^ for detecting human activity A deep net classifier and variable auto encoder were used to improve W-IoT performance. Convolutional layers were used to extract the model's latent characteristics. The Bayesian network assisted in tackling security challenges by utilising EDL and offloading. Experiments were used to prove the method's efficacy. Complex data models elevated the cost of system training beyond what was expected.

An innovative deep-learning approach for HAR was proposed by Kiran et al., (2021)^27^. First, pre-processing is used to resize video frames based on data from the target model. The pre-trained ResNet50 model is employed and trained via transfer learning in the following stage. Two succeeding layers are combined using canonical correlation analysis (CCA) after characteristics from the first layer are extracted using TL. Utilizing the Shanon Entropy approach, irrelevant data was selected to make up the fused feature vector^28^. The best classifiers are then picked based on accuracy to classify the chosen attributes using supervised learning classifiers. The results of evaluating the proposed technique using a few well-known datasets are impressive. We deduce from the accuracy that deep learning features perform better when working with large-scale datasets. It is also discovered that combining the multilayer properties produces better results. However, the effectiveness of the system is impacted by this step. The selection process thus produced more precision while simultaneously taking less time overall.

Maheswari^29^ developed a unique sparse representation theory-based technique for human action recognition utilizing video sequences. Initially, the videos were divided into numerous temporal chunks in this case. The key cuboids of interest were then extracted from the temporal segments. The histogram of Oriented Gradient (HOG) features was removed from the key cuboids. The PCA approach was used to minimize the dimension of these features. Finally, categorization was performed using the suggested Sparse Representation Modeling based Action Recognition (SRMAR) Algorithm. This method yielded great accuracy for the KTH, Olympic, and Hollywood datasets, averaging around 97.61%, 90.76%, and 73%, respectively.

Şengül^30^ describe a novel hybrid data fusion approach for forecasting three common user actions (attending a meeting, walking, and operating a motorized vehicle) using accelerometer and gyroscope data from a smartwatch connected to a mobile phone. The strategy is based on the matrix time series method for feature fusion and a modified Better-than-the-Best Fusion (BB-Fus) approach with a stochastic gradient descent algorithm to produce the best classification decision trees. Statistical pattern recognition algorithms like the Support Vector Machine (SVM) and k-Nearest Neighbour (kNN) classifiers were employed to assess user behavior. We collected and used our dataset for this investigation, which had 354 min of data from 20 subjects. We report an SVM classification accuracy of 98.32% and a kNN classification accuracy of 97.42%.

Mu et al. (2021) gave an update on previous related research and a study that included behavior representation and event modelling^31^. He offered a fresh viewpoint on event modeling by classifying the methodologies into standard event modeling, prediction model, query model, and deep hybrid model. We concluded by illuminating the datasets that are now accessible and the accepted evaluation techniques utilized in intelligent video surveillance to detect anomalous activity. More research will be performed to advance the detection of abnormal human behavior, such as weakly supervised deep generative networks. Supervision and monitoring applications in private and public spaces explicitly support and manage them.

Issa^32^ presented human Activity Recognition Based on Embedded Sensor Data Fusion for the Internet of Healthcare Things. This study assesses how well IoHT applications can be applied to a public dataset gathered using two cell phones in different positions—in the pocket and on the wrist. Three-dimensional inertia signals are produced by human activities like walking, climbing and descending stairs, writing, smoking, and other time-stamped human activities. Here is an excellent example of a human activity recognition (HAR) model. It uses Random Forest as a classifier and is built on useful handcrafted characteristics. The simulation results demonstrate that the used model is more effective for the same dataset than others suggested in the literature. Additionally, several analytic methods and implementation challenges are considered. The current model's accuracy is typically 98.7%. Further, the current model's performance is validated using data from the WISDM v1 dataset.

Şengül^33^ create a mobile application that collects and transfers acceleration and gyroscope sensor data to the cloud. We use a deep learning system in the cloud to categorize the activity according to the designated classes. We enhance the number of data samples available for training by utilizing Bica cubic Hermite interpolation, which helps the neural network's accuracy. The classifier was given the 38 statistical data features created by the rolling update method. A neural network with bidirectional long short-term memory (BiLSTM) was employed to categorize activities. The results show that when all activities are considered, our system has an accuracy of 99.59% and 97.35% in identifying falling. When only binary categorization is used, accuracy is 100%.

### Research gap

- Edge computing tries to process data locally, minimizing the need for ongoing data transmission to faraway servers. There is a gap in developing optimized algorithms and models that can evaluate sensor data effectively in real-time on wearable devices with limited resources. Finding a balance between model complexity and computing effectiveness is still challenging.
- While edge computing reduces latency by processing data locally, specific HAR applications may demand more extended processing power and storage capacities offered in the cloud. An area of investigation is bridging the gap between edge and cloud resources to enable seamless collaboration for HAR tasks.
- Small-capacity batteries often power wearable devices. A significant problem is efficiently using energy resources to perform real-time HAR without regular recharging. The research should focus on developing energy-efficient algorithms that can extend the operational life of wearable gadgets.
- HAR systems should be capable of personalizing activity recognition for specific users while maintaining their privacy. Using edge-driven solutions to balance personalization and data protection is an active field of research.
- Due to different variables, such as sensor malfunctions or environmental interference, wearable devices might acquire noisy sensor data. It is a constant problem to ensure the resilience of edge-driven HAR models in the face of such chaotic data.

## Proposed methodology for human activity recognition system

**Figure 1:**
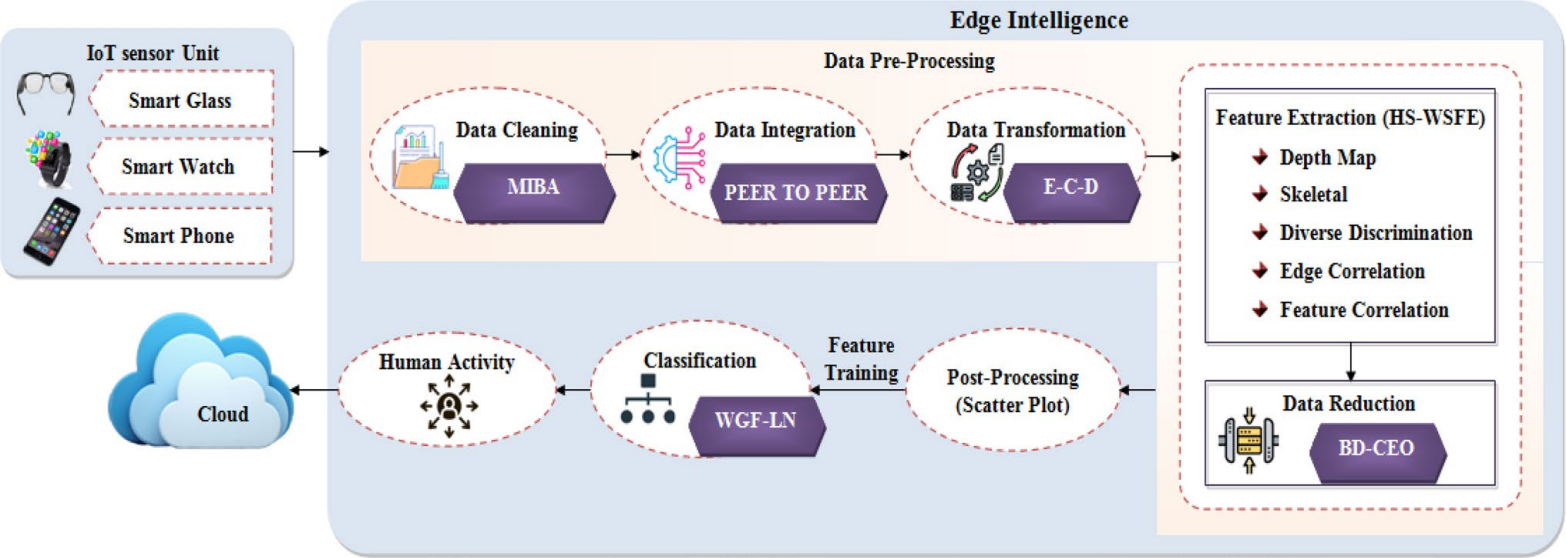
The proposed methodology is depicted as a block diagram.

Pre-processing of the data is the first step in the recommended human activity recognition system. The process then moves on to extracting the features and choosing the most crucial traits. Finally, human activities are classified based on the features chosen. Figure 1 depicts the proposed human activity recognition system's block diagram.

### Data pre-processing

The entire dataset was scaled down to 1 Hz because the data exhibited different levels of collection frequencies across other people. Scaling down decreased the amount of data to be reviewed while preserving the scope of the information and introducing consistency within the data. This step was directly tied to the study's low computation cost goal. The distinctive aspect of the data collection method was that it captured and acquired sensor data for actions that were done without moving to a new location. As a result, activities with shorter intervals have to be considered in the analysis. Because of the shorter duration, smaller window widths had to be used for feature extraction, which created a computational challenge. However, the 30-s criteria were adopted, meaning that seven activities out of 341 were removed from the dataset. As a result, data purification, integration, transformation, feature extraction, and feature reduction are performed.

#### Data cleaning

Data is originally gathered from the publically accessible dataset for this suggested edge intelligence-based human behavior recognition method. The collected data is initialized as,

1 $$ D_{n} = \left\{ {D_{1} ,D_{2} ,D_{3} ,.......D_{N} } \right\} $$

where, $D_{n}$ signifies the amount of collected data. The initialized data may contain errors such as missing values, corrupted data, duplicated data, and incorrected data. These faults happen when combining multiple data. This is corrected in the paper using data cleaning. The practice of correcting or deleting inaccurate, damaged, incorrectly formatted, duplicate, or incomplete data from a dataset is known as data cleaning. Hence, the proposed paper uses Mode-Integrated Binning Algorithm (MIBA) for data cleaning.

Data binning is a preprocessing technique used to lessen the impact of small observational errors. This technique quantifies. But, the traditional binning algorithm has a famous drawback of duplication, and thereby mode is also found to partition the database. Hence, the mode is considered when smoothing the bins by using means, medians, bin boundaries, etc. The number that appears most frequently in a data set is called the mode. Hence, in this work, the hidden value is replaced by the mode of the given values. The value that appears more frequently in a particular set of values is referred to as the mode. The mode $M \in D_{n}$ for the given data values is calculated as,

2 $$ M = l + t\frac{{\left( {F^{m} - F^{p} } \right)}}{{\left( {F^{m} - F^{p} } \right) + \left( {F^{m} - F^{s} } \right)}} $$

where, $l$ and $t$ represent the lower limit of the class interval and size of the class interval respectively, $F^{m}$ represents the frequency of the modal class, and $F^{p}$ and $F^{s}$ denote frequencies of the preceding and succeeding of the modal class. By smoothing the bins, the overall data can be obtained as,

3 $$ d_{n} = \left\{ {d_{1} ,d_{2} ,d_{3} ,.....d_{N} } \right\} $$

where, $d_{n}$ represents the number of cleaned data.

#### Data integration

Following data cleaning, the cleaned data $d_{n}$ is further given into the data integration. Data integration involves combining data residing in different sources and providing users with a unified view of them. Here, the data is integrated using peer to peer technique. A subset of techniques use disconnected sources (peers) whose pairwise schema or data-level mappings change over time and space. Combining data from numerous sources provides users and systems with more accurate information. The integrated data is denoted as $\Im$_._

#### Data transformation

After integrating the data $\Im$, data transformation is performed. This procedure divides continuous data into a collection of data intervals. For this transformation, the dataset is needed to be split into intervals of discretization. Due to this purpose, min and max levels of intervals need to be found. There is no automatic thresholding technique for finding the min and max values. This makes the operation high computing time. Hence, this paper proposes an automatic selection algorithm termed as Entropy-Candidate k partition-Discretization (E-C-D). In this automatic thresholding selection, initially, entropy is taken for the dataset and it is calculated as,

4 $$ E = C_{boltz} \ln \Im $$

where, $E$ represents entropy, and $C_{boltz}$ denotes Boltzmann constant. Following this entropy calculation, the entropy is given into the k-Means idea of merging. The K Means partition is the method of vector quantization that aims to partition the number of data into $i$ superclusters, in which each data point belongs to exactly one cluster $C_{i}$ Using the nearest mean as the standard. The algorithm partitioned the input data using the following steps such as, choosing the number of clusters, initializing centriods, assigning pixels to the nearest value, and reinitializing centroids. The intervals of discretization are splitted based on the minimum distance is calculated as,

5 $$ D = \min (\left| {C_{i} - e \in E} \right|) $$

where, $D$ represents the distance between the data point $e \in E$ and cluster $C_{i}$. Based on this above equation of minimum distance, the intervals have been splitted for the digital result $\Re$.

#### Feature extraction

Following data transformation, the features of the given input digital result $\Re$ are extracted for getting the information about the human activity with losing the original information. Hence, the key features such as depth-map function features, Skeletal features, diverse discriminatory features, edge correlation features, and correlation between features are extracted using Haar Wavelet mother- Symlet wavelet coefficient scattering feature extraction (HS-WSFE). Time–frequency analysis frequently uses the wavelet transform, which has the stability of local deformation and multi-scale. It does a good job of extracting the local feature information from signals. However, the conventional approaches have a weakness when spatial ones are overcome by complicated wavelet filters. Hence, to overcome this drawback, the mother wavelet is changed to Haar and the final filter is changed to the Symlet wavelet filter. Scale function convolution averaging, modulus operation nonlinearity, and complex wavelet transform convolution make up HS-WSFE. Each stage is described in greater detail below.

Convolution yields a locally translation-invariant digital input signal characteristic. the convolutional operator's output is,

6 $$ C_{0} \Re \left( \tau \right) = \Re *{/ \!\!\!\upsilon}_{\lambda } \left( \tau \right) $$

where, ${/ \!\!\!\upsilon}_{\lambda }$ denotes Haar wavelet with central frequency. Due to this convolution operation, the frequency information of the signal is lost. Hence, these lost frequencies are recovered by a modulus transformation $\left| {\varpi_{1} } \right|\Re$ which is calculated as,

7 $$ \left| {\varpi_{1} } \right|\Re = \left\{ {C_{0} \Re \left( \tau \right),\left| {\Re *{/ \!\!\!\upsilon}_{{\lambda_{1} }} \left( \tau \right)} \right|} \right\} $$

The first-order scattering coefficients are obtained by averaging the wavelet modulus coefficients with Symlet wavelet filter $\eta$,

8 $$ C_{1} \Re \left( \tau \right) = \left\{ {\left| {\Re *{/ \!\!\!\upsilon}_{{\lambda_{1} }} } \right|*\eta \left( \tau \right)} \right\} $$

For recovering the lost information lost by averaging, noticing $C_{1} \Re \left( \tau \right)$ can be seen as the low-frequency component of $\left| {\Re *{/ \!\!\!\upsilon}_{{\lambda_{1} }} } \right|$. Hence, the high-frequency components are extracted by using,

9 $$ \left| {\varpi_{1} } \right|\left| {\Re *{/ \!\!\!\upsilon}_{{\lambda_{1} }} } \right| = \left\{ {C_{1} \Re \left( \tau \right),\left| {\Re *{/ \!\!\!\upsilon}_{{\lambda_{1} }} |*{/ \!\!\!\upsilon}_{{\lambda_{2} }} \left( \tau \right)} \right|} \right\} $$

The above equation further derives the second-order coefficient $C_{2} \Re \left( \tau \right)$ by,

10 $$ C_{2} \Re \left( \tau \right) = \left\{ {|\left| {\Re *{/ \!\!\!\upsilon}_{{\lambda_{1} }} |*{/ \!\!\!\upsilon}_{{\lambda_{2} }} } \right|*\eta \left( \tau \right)} \right\} $$

The above iterations define the wavelet modulus convolutions $\kappa_{n} \Re \left( \tau \right)$ as,

11 $$ \kappa_{n} \Re \left( \tau \right) = \left\{ {|\left| {\left| {\Re *{/ \!\!\!\upsilon}_{{\lambda_{1} }} } \right|*.........|*{/ \!\!\!\upsilon}_{{\lambda_{n} }} } \right|} \right\} $$

After calculating the convolution till the $n{\rm th}$ order, scattering coefficients are calculated by averaging $\kappa_{n} \Re \left( \tau \right)$ with $\eta \left( \tau \right)$ is expressed as,

12 $$ C_{n} \Re \left( \tau \right) = \left\{ {|\left| {\left| {\Re *{/ \!\!\!\upsilon}_{{\lambda_{1} }} } \right|*.........|*{/ \!\!\!\upsilon}_{{\lambda_{n} }} } \right|*\eta \left( \tau \right)} \right\} $$

By summing all the defined scattering, coefficients in each order can be obtained as overall scattering coefficients. Through this process of defining coefficients, the important features of digital results can be obtained and it is expressed as,

13 $$ \rho_{m} = \left\{ {\rho_{1} ,\rho_{2} ,\rho_{3} ,.....\rho_{M} } \right\} $$

where, $\rho_{m}$ denotes the no. of extracted features. The pseudo-code of the proposed HS-WSFE is,


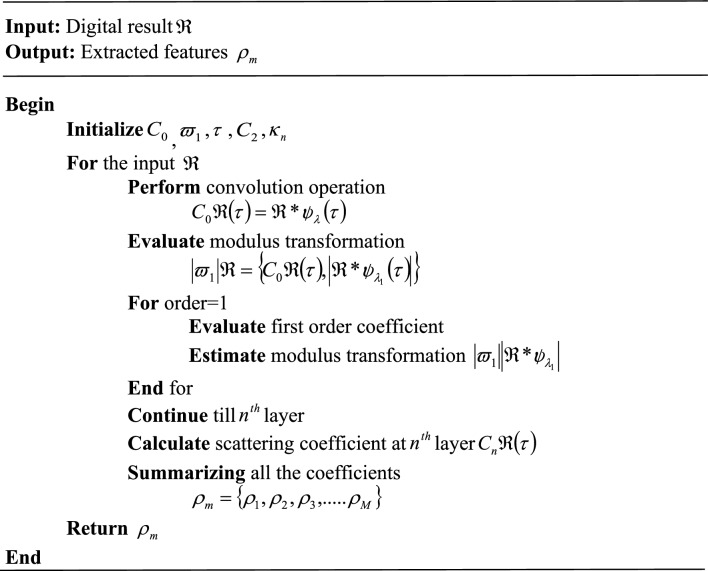


Benefits of Haar Wavelet mother- Symlet wavelet coefficient scattering feature extraction (HS-WSFE):

- The approach captures sudden shifts and complex patterns in motion data by combining the strengths of Haar and Symlet wavelets, resulting in more discriminative features.
- The edge-detection capabilities of Haar Wavelet boost the system's capacity to recognize activities with sharp transitions.
- Symlet wavelet coefficient scattering improves the system's resistance to tiny fluctuations in motion patterns, making it ideal for real-world scenarios.
- The technique is tailored for wearable devices, assuring compatibility with their computational resource and battery consumption limits.

The integration of edge-driven intelligence based on HS-WSFE presents a promising option for increasing human activity identification utilizing wearable devices. The precision and application of HAR systems may be improved by the method's capacity to extract pertinent features from motion data in various situations. As wearable technology advances, unique feature extraction techniques such as HS-WSFE may pave the way for more sophisticated and effective human activity recognition solutions.

#### Feature reduction

In this section, the important features are selected from the extracted features $\rho_{m}$ using Binomial Distribution integrated-Golden Eagle Optimization (BD-GEO). A new meta-heuristic method called Golden Eagle Optimization (GEO) was developed in response to the spiraling foraging behavior of flying raptors. to successfully finish the cruise vector, as well as exploration and exploitation. But, the traditional methods have unstable convergence in the non-continuous decision space. Hence, the plane space and step factor are changed to follow Binomial Distribution. The mathematical expression for the hunting strategy is explained below,

Here, the extracted features are considered as the number of the golden eagle and then the populations are initialized. For calculating the fitness, the vectors are found for the sigma value. Then, the vectors are compared with adaptive angle sigma if smaller than iterated or if greater than reduced dimensional values.

##### (a) Prey selection

In this step, each eagle selected its prey by memorizing the best solution. Then, the attack and cruise procedures are carried out on the selected prey.

##### (b) Attack

The golden eagle launches a vector assault, moving in a straight line from its current location to the most recent spot where it was able to watch its target. Because of the vector, a great number of eagles can be gathered and employed in an attack. Technique of attack for $i{\rm th}$ golden eagle $\overrightarrow {{\alpha_{i} }}$ can be expressed mathematically as,

14 $$ \overrightarrow {{\alpha_{i} }} = \overrightarrow {P} - \overrightarrow {{\chi_{{_{i} }} }} $$

where, $\overrightarrow {P}$ The most ideal place the eagle has discovered so yet. $\rho_{i}$, and $\overrightarrow {{\chi_{{_{i} }} }}$ indicates the eagle's location at the moment in question $\chi$. form $s$.

##### (c) Cruise

In this instance, the attack vector determines the cruise vector. Instead of running parallel to the circle, the cruise vector is perpendicular to it. Solving for the scalar form $s$.

of the space-bound tangent hyperplane yields the cruise vector.

15 $$ s = \sum\limits_{j = 1}^{q} {\wp_{j} v_{j} } = \sum\limits_{j = 1}^{q} {\beta_{j} } x_{j} $$

where, $\wp_{j}$ and $v_{j}$ represents normal vector and variables vector,$j$ signifies dimensional space, $\beta_{j}$ denotes $j{\rm th}$ consider an attack's path $\overrightarrow {{\alpha_{i} }}$, and $x_{j}$ signifies the location of the selected prey. Following the calculation of scalar form, the below steps are carried out for finding the location of a golden eagle.

- Initially, one random variable is selected from the list of the random variable for serving as a fixed variable $\hbar$.
- Give arbitrary values to all variables except the fixed one.
- Determine which element is a constant $\hbar$
  16 $$ A_{\hbar } = \frac{{s - \sum\limits_{j;j \ne \hbar } {\beta_{j} } }}{{\beta_{\hbar } }} $$

Where, $A_{\hbar }$ signifies $\hbar{\rm th}$ element of the destination point $A$, and $\beta_{\hbar }$ is the $\hbar{\rm th}$ element of the attack vector. The random destination point on the cruise hyperplane is displayed in the general representation as,

17 $$ \overrightarrow {{G_{i} }} = \left( {A_{1} = random,A_{2} = random,........,A_{\hbar } = A_{\hbar } = \frac{{s - \sum\limits_{j;j \ne \hbar } {\beta_{j} } }}{{\beta_{\hbar } }},.....,A_{m} = random\,} \right) $$

From this destination point, The Golden Eagle's cruise vector is iterated.$\left( {iter} \right)$.

##### (d) Movement toward new positions

After finding the destination point, the eagle moves toward the prey. The displacement comprises both the vector and the assault We'll look at the golden eagle's step vector in the following section $\left( {iter} \right)$ is expressed as,

18 $$ \wp_{i} = \overrightarrow {R}_{1} k_{attack} \frac{{\overrightarrow {{\alpha_{i} }} }}{{\left\| {\overrightarrow {{\alpha_{i} }} } \right\|}} + \overrightarrow {R}_{2} k_{cruise} \frac{{\overrightarrow {{G_{i} }} }}{{\left\| {\overrightarrow {{G_{i} }} } \right\|}} $$

Here, $k_{attack}$ and $k_{cruise}$ are the attack and cruise coefficients,$\wp$ represents step vector, and $\overrightarrow {R}_{1}$ and $\overrightarrow {R}_{2}$ denote random vectors, which are calculated as,

19 $$ u = \left( \begin{gathered} w_{1} \hfill \\ w_{2} \hfill \\ \end{gathered} \right)\Psi^{{w_{1} }} \nabla^{{w_{2} - w_{1} }} \,\,\,\,where,u = R_{1} ,R_{2} $$

Here, $u$ is the output of the binomial distribution, $w_{1}$ represents the number of trials, $w_{2}$ denotes The frequency with which a particular outcome occurred during the trials determines the success rate, failure rate, and overall success rate for each trial $\left\| {\overrightarrow {{\alpha_{i} }} } \right\|$ and $\left\| {\overrightarrow {{G_{i} }} } \right\|$ are the Euclidean norms, which are derived by using the equation is,

20 $$ \left\| {\overrightarrow {{\alpha_{i} }} } \right\| = \sqrt {\sum\limits_{j = 1}^{q} {\beta_{j}^{2} } } $$

21 $$ \left\| {\overrightarrow {{G_{i} }} } \right\| = \sqrt {\sum\limits_{j = 1}^{q} {A_{j}^{2} } } $$

The step vector in the next iteration $\left( {\wp^{{\left( {iter} \right) + 1}} } \right)$ is calculated as,

22 $$ \wp^{{\left( {iter} \right) + 1}} = \wp^{{\left( {iter} \right)}} + \wp_{i}^{{^{{\left( {iter} \right)}} }} $$

The iteration increases the attack mode while decreasing the cruise.

##### (e) Transition from cruise to attack

Continuing the iteration is helpful for hunting the prey. In order this, transition of the golden eagle prey is calculated as,

23 $$ \left\{ \begin{gathered} k_{attack} = \left( {k_{attack} } \right)^{0} + \frac{iter}{{\left( {iter} \right)^{\max } }}\left| {\left( {k_{attack} } \right)^{{\left( {iter} \right)^{\max } }} - \left( {k_{attack} } \right)^{0} } \right| \hfill \\ k_{cruise} = \left( {k_{cruise} } \right)^{0} + \frac{iter}{{\left( {iter} \right)^{\max } }}\left| {\left( {k_{cruise}^{0} } \right)^{{^{{\left( {iter} \right)^{\max } }} }} - \left( {k_{cruise} } \right)^{0} } \right| \hfill \\ \end{gathered} \right. $$

Here, $\left( {k_{attack} } \right)^{0}$ and $\left( {k_{cruise} } \right)^{0}$ are the initial the propensity to attack and cruise, and $\left( {k_{attack} } \right)^{{\left( {iter} \right)^{\max } }}$ and $\left( {k_{cruise}^{0} } \right)^{{^{{\left( {iter} \right)^{\max } }} }}$ represents the propensity to attack's final values to attack and cruise. In this same way of transiting procedure, the important features are selected and it is expressed as,

24 $$ r^{b} = \left\{ {r^{1} ,r^{2} ,r^{3} ,.....r^{\nabla } } \right\},\,\,where,\,\phi = 1,2,3,......\nabla $$

where, $r^{b}$ denotes number of selected features.

Benefits for Binomial Distribution integrated Golden Eagle Optimization (BD-GEO)

- The suggested method helps identify and retain the essential data for recognizing human activity by fusing Binomial Distribution with Golden Eagle Optimization. This leads to decreased misclassification, greater accuracy when categorizing various activities, and more trustworthy outcomes.
- BD-GEO improves computational efficiency by combining the strengths of Binomial Distribution and Golden Eagle Optimization. Because of this synergy, the technique can reach an optimal feature subset faster, decreasing the computational cost and making it ideal for real-time applications on resource-constrained wearable devices.
- Overfitting is reduced since the integration of Binomial Distribution considers the significance of each feature based on its contribution to overall recognition performance. This keeps the model from being overly personalized to the training data and guarantees that it generalizes to new data instances more effectively.
- The approach's Edge Driven Intelligence feature is very beneficial for wearable devices. The need for constant data transfer to a central server for processing is avoided by performing feature reduction directly on the wearable device. This results in faster response times and lower latency, making it ideal for real-time monitoring and feedback.
- Reducing features at the edge reduces the quantity of data that must be transferred and processed on remote servers. This data transmission and processing reduction significantly reduces wearable device energy consumption, prolonging battery life and allowing for more extended usage.
- Due to various causes, wearable devices frequently face noisy and incomplete sensor data. The Binomial Distribution-guided feature reduction technique aids in picking features that are resistant to noise, ensuring that the model concentrates on the most informative portions of the data.

BD-GEO, for feature reduction using Binomial Distribution integrated-Golden Eagle Optimization, in conjunction with the HS-WSFE framework, to improve wearable devices' human activity identification capabilities. The suggested method exhibits its ability to pick essential characteristics effectively and improve sensor fusion, enhancing recognition accuracy while minimizing computational complexity. This study advances wearable-based activity recognition systems, paving the way for more successful applications in healthcare, sports, and other fields.

### Post processing

In this section, the selected features are post-processed, which means normalized within the range of the variation between the features. Here, the post-processing is done using a scatter plot to matrix technique A scatter-plot matrix combines the results of numerous scatter plots with related variables. Cartesian co-ordinates are used to plot the feature points on the graph. By combining all these feature points, a single appropriate graph is built. It shows the correlation of all the feature points and is helpful to train the classifier. The scatter matrix $\delta_{b} \in r^{b}$ is computed by the following equation is,

25 $$ \delta_{b} = \sum\limits_{\phi = 1}^{\nabla } {\left( {r^{\phi } - \aleph } \right)} \left( {r^{\phi } - \aleph } \right)^{T} $$

26 $$ \aleph = \frac{1}{\nabla }\sum\limits_{\phi = 1}^{\nabla } {r^{\phi } } $$

where, $r^{\phi }$ is the $\phi{\rm th}$ feature, and $\aleph$ represents the mean vector of $r^{\phi }$.

### Classification

**Figure 2:**
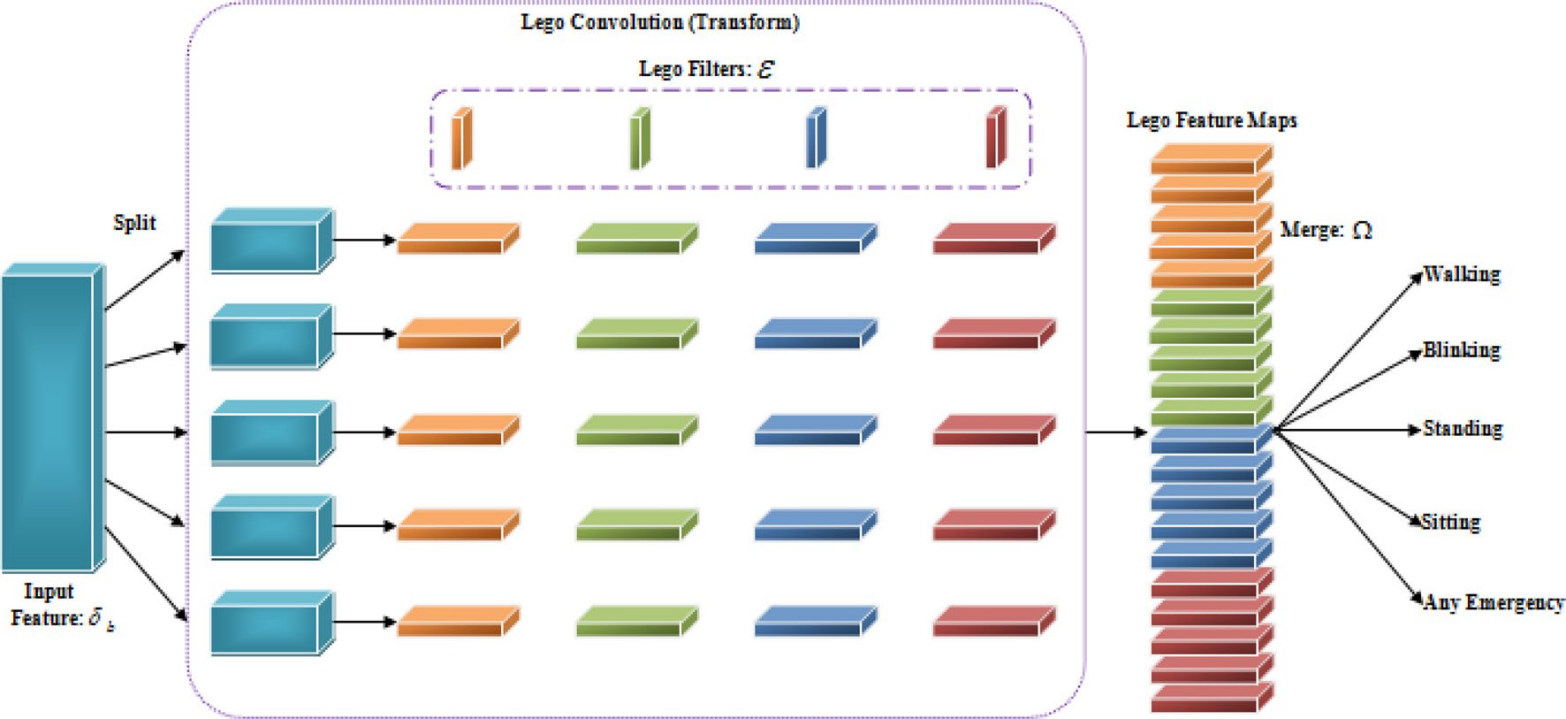
WGF-LN basic architecture.

In this phase, the post-processed features $\delta_{b}$ are transferred into the wasserstein gradient flow legonet (WGF-LN). In general, LegoNets are efficient convolutional neural networks constructed with Lego filters. The network works based on the strategy of the split-transform merge to accelerate their convolutions. The inability of traditional legonet memory to be reused due to backward locking prevents the adoption of wearable activity recognition systems. For wearable activity recognition to be practical, this constraint, as well as local losses, must be addressed and therefore the estimator is changed to wasserstein gradient flow estimator. The proposed structure of WGF-LN is displayed in Fig. 2.

As per the strategy, at first, the input features are splitted into a number of fragments. The fragments are convolved with each individual Lego filter. The convolution operation $\gamma$ can be expressed as,

27 $$ \gamma = \delta_{b} \left( {\varepsilon \Omega } \right) $$

where, $\Omega$ represents vector mask for selecting only one lego filter, and $\varepsilon$ denotes lego filters. For $\iota{\rm th}$ output, $\gamma_{\iota }$ can be written as,

28 $$ \gamma_{\iota } = \sum\limits_{\phi = 1}^{\nabla } {\delta_{\phi }^{T} } \left( {\varepsilon \Omega_{\iota }^{\phi } } \right) $$

Here, $\delta_{\phi }$ represents $\phi{\rm th}$ input feature, and $\Omega_{\iota }^{\phi }$ specifies a vector mask for $\phi{\rm th}$ input feature at $\iota{\rm th}$ the convolution layer. From the perspective of the matrix, $\gamma_{\iota }$ can be expressed as,

29 $$ \gamma_{\iota } = \sum\limits_{\phi = 1}^{\nabla } {\left( {\delta_{\phi }^{T} \varepsilon } \right)} \Omega_{\iota }^{\phi } $$

where, $\delta_{\phi }^{T} \varepsilon$ denotes the intermediate logo feature map $\vartheta$.$\Omega$ Selects feature maps from $\vartheta$. Finally, the output can be obtained by merging all the selected feature maps. It is calculated as,

30 $$ \gamma_{n} = - {\rm Z}\left( {\gamma_{\iota } } \right) $$

where, ${\rm Z}$ represents the merging function, which is done by to wasserstein gradient flow estimator, and $\gamma_{n}$ is the output of the human activity. The final output exposed human activity such as walking, blinking, standing, sitting, and any emergency. At last, the loss function is decided by comparing the obtained output to the desired output. and it is calculated as,

31 $$ \varphi_{loss} = \left( {\gamma_{n} ,\Upsilon } \right) $$

where, $\varphi_{loss}$ is the loss function, and $\Upsilon$ denotes ground truth. If there is a loss happened, the same operation is iterated. Otherwise, the operation is terminated. The activities are finally uploaded to the cloud. The pseudo-code of the proposed WGF-LN is,


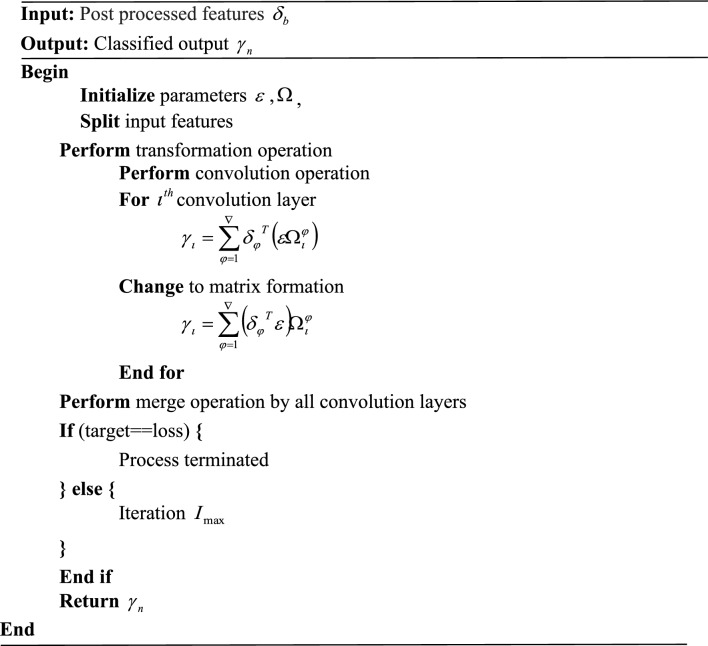


## Result and discussion

In this section, the model's performance will be analyzed and contrasted with that of other methodologies presently in use, demonstrating the model's utility. The experiment was implemented on the platform of *JAVA*.

Effectiveness of proposed methodology:

- Using a state-of-the-art approach like "WGF-LN-based edge-driven intelligence," the field of human activity recognition may have acquired a new perspective. The novelty of the method might take time to be apparent in the experimental data, but it might present a fresh and exciting direction for further study.
- New approaches frequently have a higher learning curve for both researchers and reviewers. The approach's complexity may have resulted in specific experimental problems, such as parameter adjustment, data preprocessing, or implementation complexities, which could have influenced the original results. Researchers may discover methods to improve the process with more time and refining.
- The quantity and quality of the dataset used for training and efvalidauation can impact the success of a new methodology. The model's performance may not correctly reflect its real potential if it was trained on a small or unrepresentative dataset. Increasing the dataset's size or diversity may yield better results.
- Machine learning models frequently require careful tuning of hyperparameters to obtain optimal performance. The model's effectiveness could have been jeopardized if the hyperparameters were not been properly tweaked during the initial experiments. These parameters could be tweaked to improve performance.
- Scientific research is frequently iterative. The preliminary results can act as a springboard for further investigation and development. Researchers can improve the current approaches by tweaking algorithms, adding input, and fixing limitations found during the initial testing.

### Dataset description

Public datasets encourage collaboration and accountability for published work, among other advantages. Associations have been made in affective computing, athletic activity recognition, and the research of HAR for personal health monitoring. Users are accountable for their published work because it can be reproduced and verified by others. To assist brand-new HAR users, a few publicly accessible datasets are offered and explained below. Popular public datasets have been used in the literature under review, including the 2012-released UCI Public Domain Dataset for HAR. There are rudimentary activity statistics for standing, sitting, lying down, and walking. A list of the HAR public datasets is provided in Table 2, along with a summary of each dataset. In other words, training uses 80% of the dataset, and testing uses 20%.

- The 2014-created mHealth dataset has 12 activities, three sensors, and ten persons. Vital signs and bodily mobility were both monitored during the experiment. Sensors were put on the chest, right wrist, and left ankle to record motions. A 2-lead ECG measurement is also taken for essential monitoring.
- A dataset called PAMAP2, with 18 activities, was released in 2012. Four different sensors were used, and only nine participants were examined. The heart rate monitor in this dataset distinguishes it. The difficulty of the workouts, which included walking, cycling, and soccer, was measured using a heart rate monitor. Analyses from the heart rate monitor may expand affective computing.
- The WISDM dataset became available in 2019. This dataset contains a raw accelerometer and gyroscope sensor data from a smartwatch and a smartphone. There are 18 actions and 51 persons in the dataset. Even though this dataset only includes an accelerometer, behavioral biometric models can still be made with it. The research using this information showed how useful motion-based biometrics are. Since categorizing activity recognition is the main objective of the initial evaluation, the 2-lead ECG is not utilized. Future studies on affective computing, exercise intensity, and patient health monitoring can all benefit from using ECG data.
- In 2013, the public HAR dataset became available. It contains inertial data gathered from the accelerometer and gyroscope of a smartphone. The dataset aims to categorize six distinct human activities. The HAR experiment's action steps are shown in a movie on the public repository. A 50 Hz sample frequency was used to record 30 participants for the database. Since its release in 2013, this dataset has been the focus of substantial research.

**Table 2:** Public datasets list.

Dataset	Modalities of sensors	Number of sensors	# of participants	Activities
w-HAR (Bhat et al., 2020)^41^	A stretch sensor and an accelerometer	2	22	Jump, stand, and down the stairs, Walk, move upstairs, sit, and rest between chores
mHealth (Banos et al., 2015)^42^	Electrocardiogram, magnetometer, gyroscope, and accelerometer	4	10	Walking, climbing stairs, bending forward at the waist, raising the arms in front of the body, bending the knees, cycling, jogging, running, and hopping along and backward are all examples of static poses
PAMAP2 (Reiss & Stricker, 2012)^43^	Gyroscope, magnetometer, and accelerometer	4	18	Rope jumping, lying, sitting, standing, Nordic walking, housecleaning, computer work, driving, stair ascending, stair descending, vacuuming, ironing, folding clothing, and soccer
WISDM v2 (Weiss et al., 2019)^44^	Accelerometer	1	29	Walking, running, stair climbing, sitting, and standing
Public HAR (Anguita et al., 2013)^45^	Accelerometer and gyroscope	1	30	Moving from one level to another, walking, sitting, standing, and lying

### Performance analysis of data cleaning

This section provides the results about the efficiency of the data cleaning method. Analyses and comparisons of MIBA and its evaluation are made with other approaches. Binning algorithms, EFB, and EWB are some of the current techniques.

**Figure 3:**
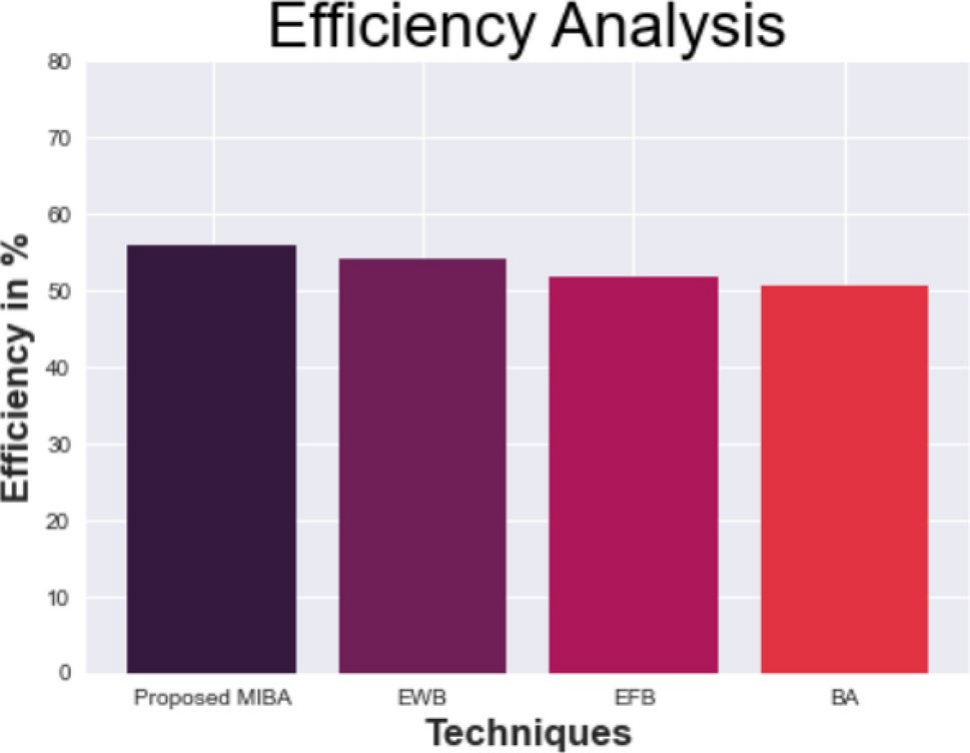
The performance of the proposed and existing model based on the efficiency.

The effectiveness of the suggested and standard procedures is shown in Table 3 and Fig. 3. Here, the efficiency is calculated using the accurate correction of data inconsistencies and outlier detection. The efficiency of the modern way triumphs over the existed method. The efficiency of the proposed model achieved 56%, which shows a 2.01% improvement from existing EWB, 4.24% improvement from EFB, and 5.49% improvement from BA. From the entire comparison, The plan appears to be working very well than the existing methods.

**Table 3:** Comparison of the proposed model to existing methodologies.

Techniques	Efficiency
Proposed MIBA	56
EWB	53.99
EFB	51.76
BA	50.51

### Performance analysis of feature extraction

The suggested HS-WSFE has been tested against existing models such as AutoEncoder (AE), Deep Neural Networks (DNN), and Wavelet Scattering (WS) to show that it is both practical and efficient.

**Figure 4:**
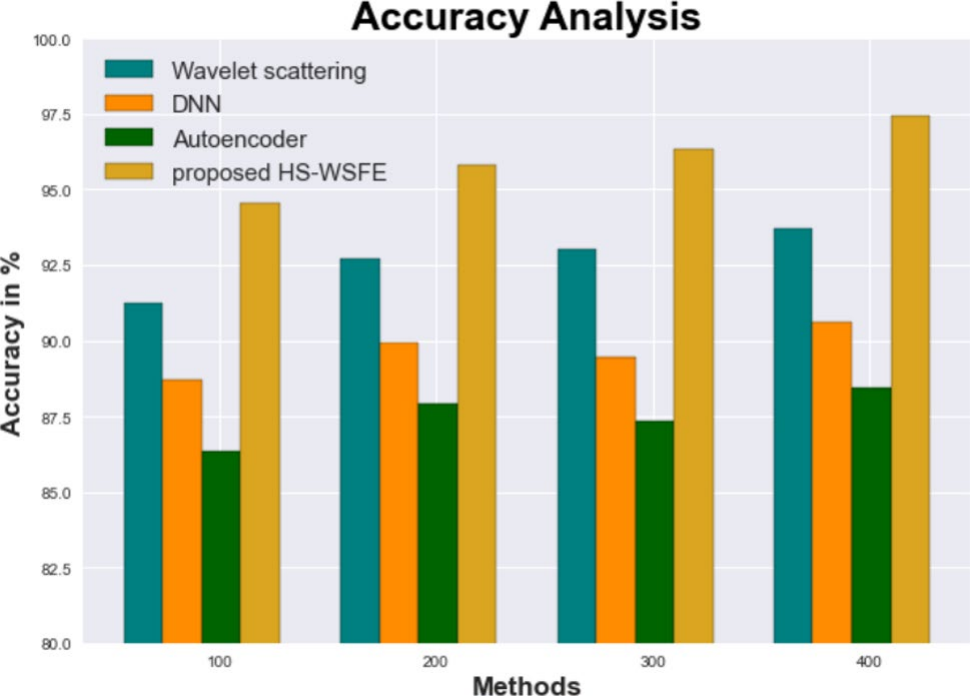
The performance of the proposed and existing model based on the Accuracy.

Figure 4 and Table 4 illustrate Comparison of the HS-WSFE results with other commonly used techniques The graph shows how DL the Wavelet scattering ,DNN , Auto encoder models have obtained accuracy of 91.228%, 88.725% and 86.373%, respectively. However, the HS-WSFE In tests using datasets of various sizes, the model consistently outperformed its competitors. HS-WSFE, like HS-WSFE, has an accuracy value of 97.435% when applied to less than 400 data points while it is 93.726%, 90.627% and 88.435% for Wavelet scattering, DNN, Auto encoder models, respectively.

**Table 4:** The performance of the proposed and existing model based on the accuracy.

No of data from dataset	Wavelet scattering	DNN	Autoencoder	Proposed HS-WSFE
100	91.228	88.725	86.373	94.536
200	92.735	89.927	87.938	95.836
300	93.028	89.424	87.324	96.335
400	93.726	90.627	88.435	97.435

### Performance analysis of feature reduction

The performance of the suggested BD-GEO is examined in this section together with that of many other techniques, such as the Golden Eagle Optimizer (GEO), Grey Wolf Optimizer (GWO), Genetic Algorithm (GA), and Particle Swarm Optimization (PSO).

**Figure 5:**
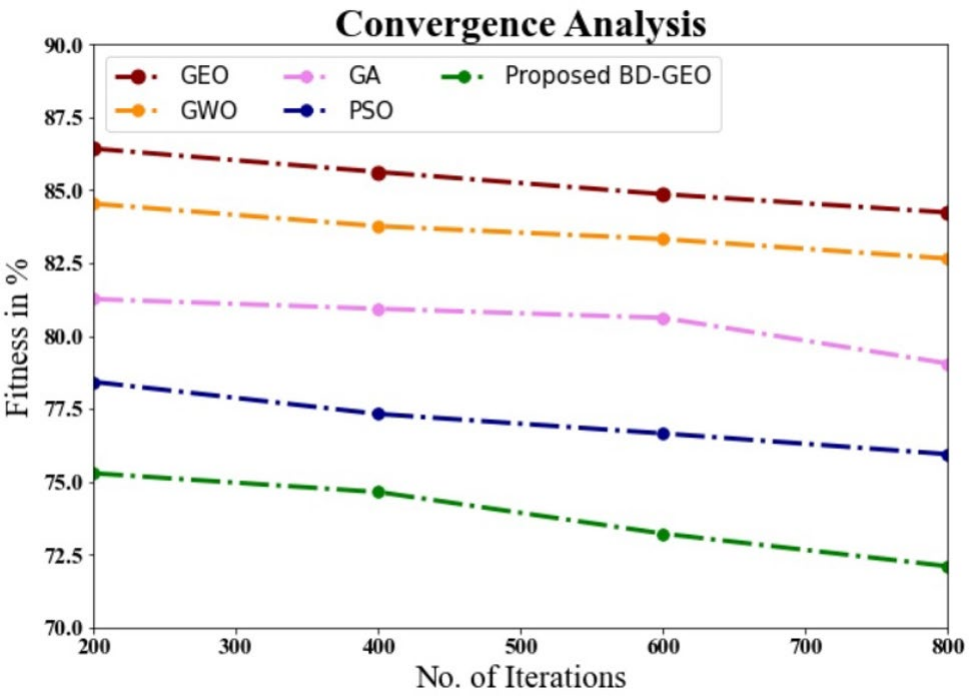
Convergence Analysis.

Figure 5 and Table 5 compare the BD-Convergence GEO to other techniques. Convergence's performance has improved as shown in the graph, thanks to DL. In the instance of data set 200, the BD-accuracy GEO is 75.287%, while the convergence values for the GEO, GWO, GA, and PSO models are 86.425%, 84.535%, 81.264%, and 78.422%, respectively. The BD-GEO model performs well with data sets of varied sizes. In comparison to GEO, GWO, GA, and PSO, which have values of 84.234%, 82.654%, 79.054%, and 75.943%, respectively, BD-GEO under 800 data has a convergence value of 72.098%.

**Table 5:** Convergence analysis for BD-GEO methos with existing systems.

Iterations	GEO	GWO	GA	PSO	Proposed BD-GEO
200	86.425	84.535	81.264	78.422	75.287
400	85.624	83.765	80.932	77.322	74.643
600	84.862	83.322	80.621	76.654	73.216
800	84.234	82.654	79.054	75.943	72.098

### Performance analysis of classification

This section compares and contrasts the proposed WGF-LN's performance with those of existing networks such as LegoNet, Convolutional Neural Network (CNN), and Deep Neural Network (DNN). The metrics for the analysis include f-score, recall, accuracy, and precision. True positive (TP) and true negative (TN), which indicate correctly identified emotional states, and false positive (FP) and false negative (FN), which indicate incorrectly identified emotional states, are two of these metrics, which are typically calculated based on four key metrics of a binary classification outcome (positive/negative). The following is a list of some performance indicators.

Accuracy: This statistic gauges the frequency of correctly identified cases. If the classes are balanced, or each class has an equal number of samples, the system operates effectively. Utilizing Eq. (32) to calculate it

32 $$ Accuracy = \frac{TP + TN}{{TP + FN + TN + FP}}*100 $$

Precision: This measure shows the proportion of correctly classified items. Using Eq. (33), it can be indicated.

33 $$ \Pr ecision = \frac{TP}{{TP + TP}}*100 $$

Recall: Recall or true positive rate are other names for it. It assesses how frequently a classifier classifies a successful outcome correctly. Equation (34) is described.

34 $$ {\text{Re}} call = \frac{TP}{{TP + FN}}*100 $$

F-Measure: The harmonic mean represents precision and recall. It is significant since increased precision leads to decreased recall and vice versa. Equation (35) calculates it.

35 $$ F{ - }Score = 2*\frac{{\Pr ecision*{\text{Re}} call}}{{\Pr ecision + {\text{Re}} call}} $$

#### Precision

**Figure 6:**
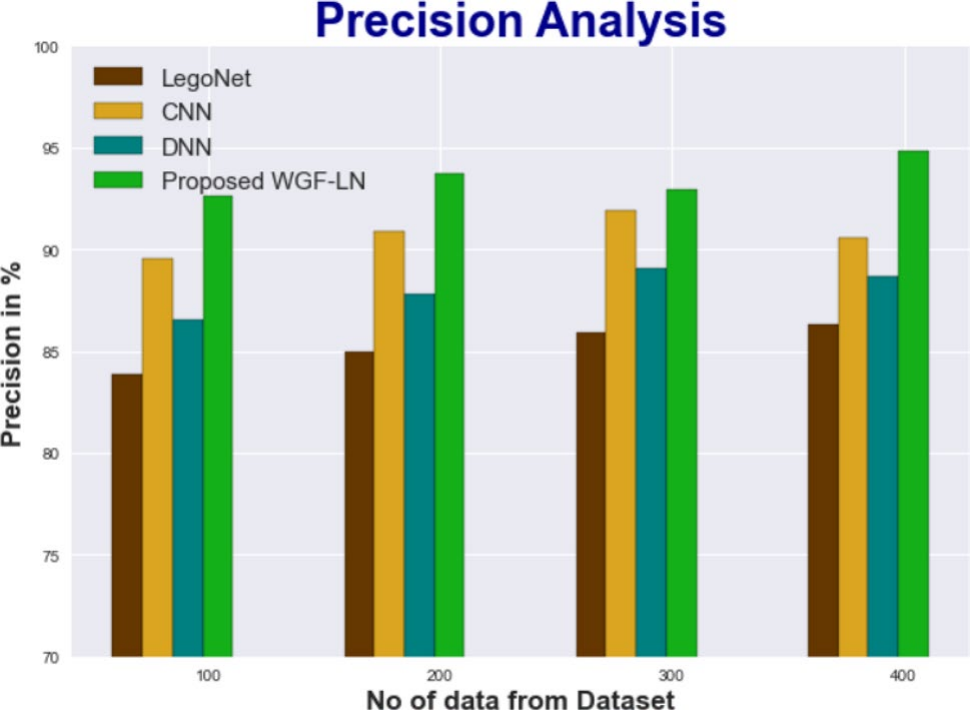
Precision analysis for WGF-LN technique with existing systems.

The findings of a detailed comparison of the WGF-LN method to other existing methodologies are displayed in Fig. 6 and Table 6. The figure demonstrations that precision has increased after implementing DL. When applied to data set 100, WGF-LN achieves a precision of 92.635%, while LegoNet, CNN, and DNN models achieve 83.847%, 89.534%, and 86.536%, respectively. The WGF-LN model has shown its best performance across a range of dataset sizes. WGF-LN under 400 data has a precision value of 94.857%, while LegoNet, CNN, and DNN models are 86.324%, 90.533%, and 88.633%.

**Table 6:** Precision analysis for WGF-LN technique with existing systems.

No of data from dataset	LegoNet	CNN	DNN	Proposed WGF-LN
100	83.847	89.534	86.536	92.635
200	84.937	90.856	87.836	93.745
300	85.923	91.873	89.102	92.947
400	86.324	90.533	88.633	94.857

#### Recall

**Figure 7:**
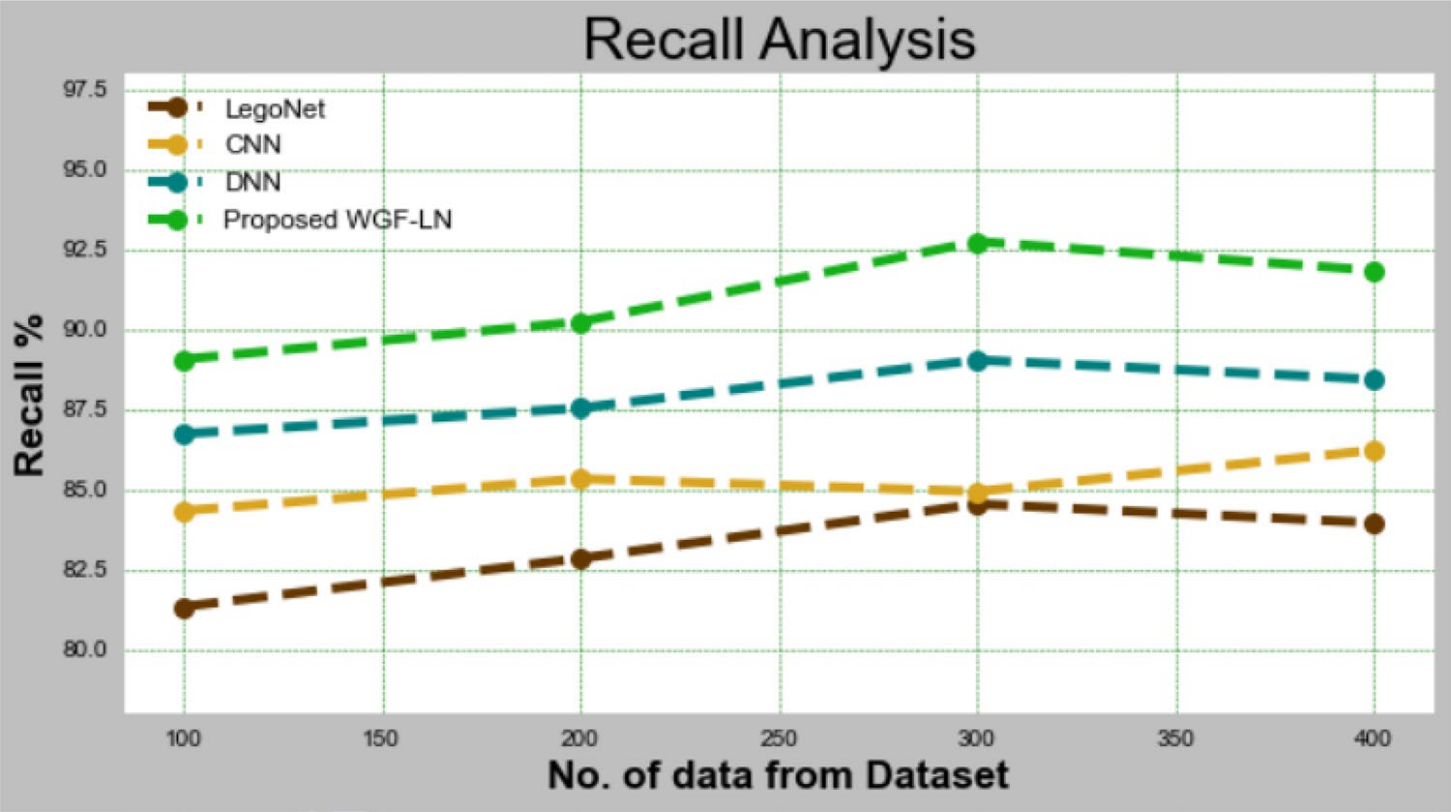
Recall analysis for WGF-LN technique with existing systems.

The recall of the WGF-LN methodology is associated to that of other methodologies in Fig. 7 and Table 7. The graphic displays how DL has enhanced recall. With data set 100, WGF-recall LNs had an accuracy of 89.052%, compared to 81.325%, 84.326%, and 86.735% for LegoNet, CNN, and DNN models, respectively. Large data sets allow the WGF-LN model to function well. Under 400 data points, the recall values for LegoNet, CNN, and DNN models were all similar to WGF-LN, at 83.947%, 86.224%, and 88.42%, respectively.

**Table 7:** Recall analysis for WGF-LN technique with existing systems.

No of data from dataset	LegoNet	CNN	DNN	Proposed WGF-LN
100	81.325	84.326	86.735	89.052
200	82.836	85.325	87.526	90.225
300	84.536	84.926	89.027	92.735
400	83.947	86.224	88.425	91.832

#### F-Score

**Figure 8:**
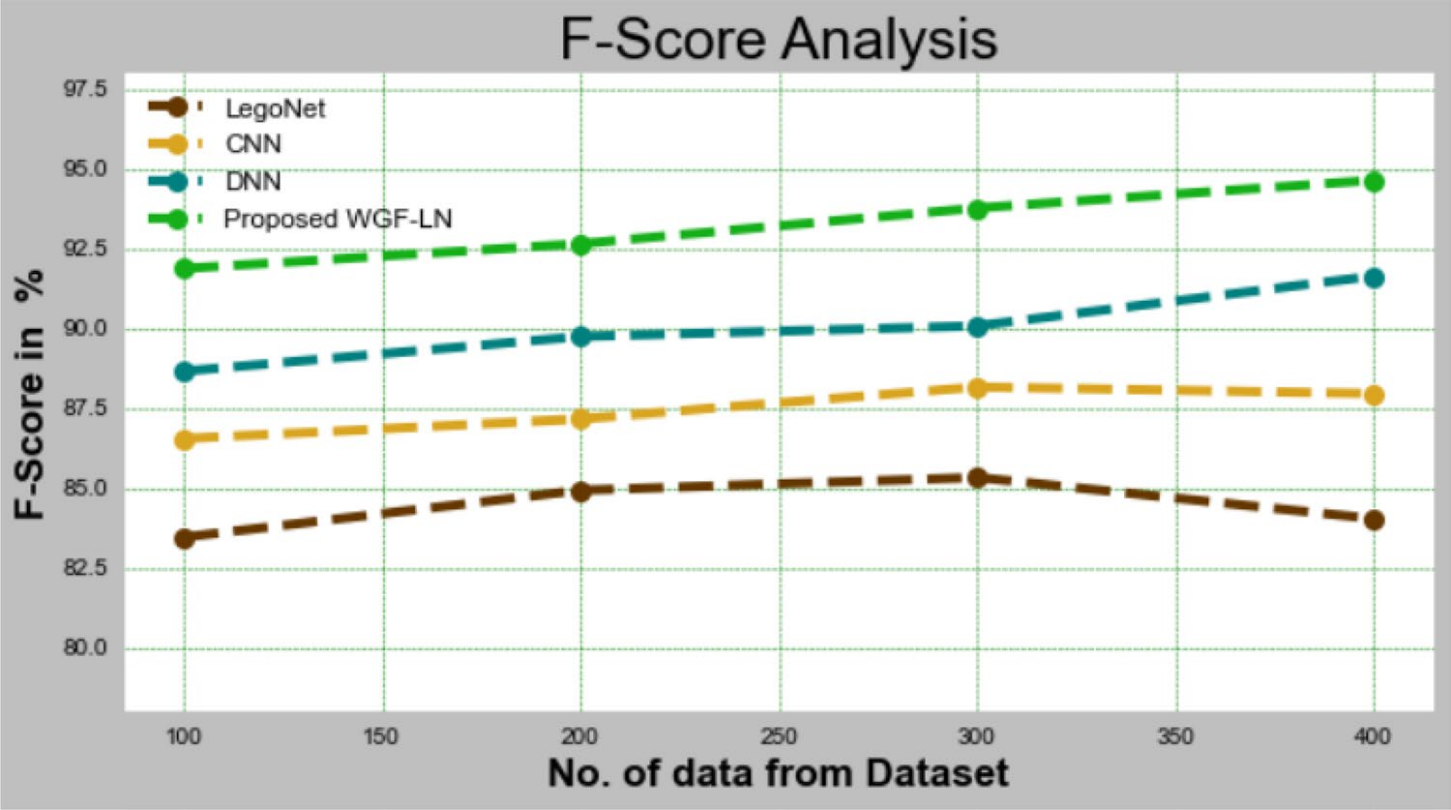
F-Score analysis for WGF-LN method with existing systems.

WGF-f-score LNs are contrasted to other methods in Fig. 8 and Table 8. DL improved f-score performance, as shown in the graph. LegoNet, CNN, and DNN received 83.452%, 86.543%, and 88.643% of the WGF-f-score LNs with data set 100, respectively. Large data sets allow the WGF-LN model to perform well. The f-scores of the LegoNet, CNN, and DNN models are 84.029%, 87.943%, and 91.632%, respectively, which are comparable to the WGF-LN under 400 data.

**Table 8:** Analysis of F-Scores for the WGF-LN approach with existing systems.

No of data from dataset	LegoNet	CNN	DNN	Proposed WGF-LN
100	83.452	86.543	88.643	91.864
200	84.923	87.154	89.732	92.643
300	85.324	88.146	90.065	93.764
400	84.029	87.943	91.632	94.635

#### Accuracy

**Figure 9:**
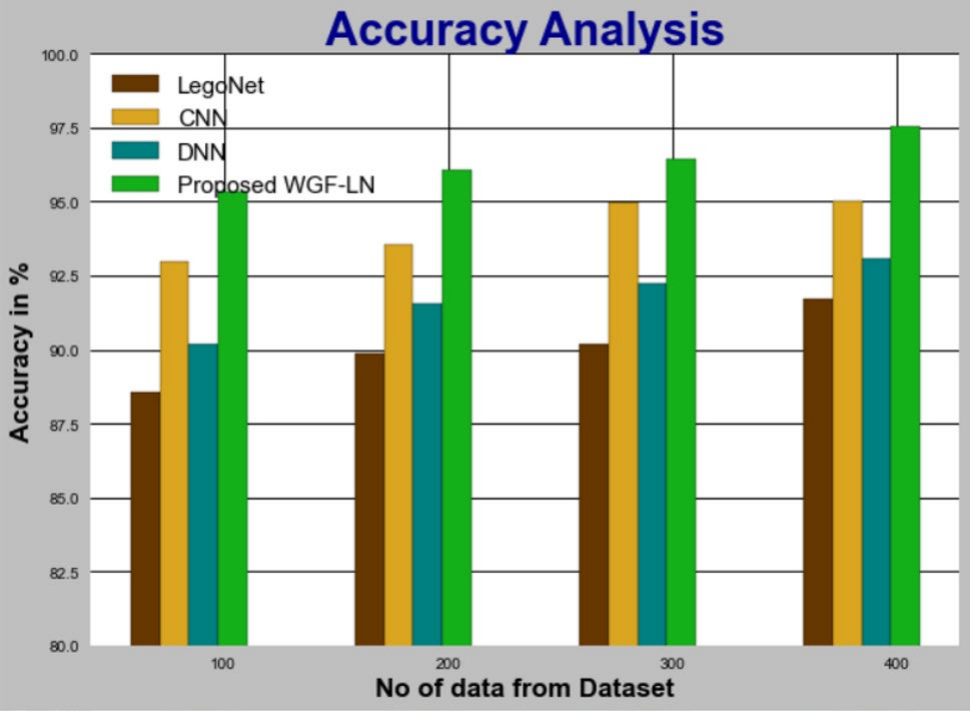
Accuracy analysis for WGF-LN method with existing systems.

The WGF-LN methodology is compared to other methodologies in Fig. 9 and Table 9. The figure shows how DL improves accuracy. The accuracy of WGF-LN with data set 100 is 95.329%, compared to 88.532%, 92.946%, and 90.165% for the LegoNet, CNN, and DNN models, respectively. The WGF-LN model performs well with large data sets. WGF-LN under 400 data accuracy is 91.721%, 93.726%, and 95.032% when compared to LegoNet, CNN, and DNN models.

**Table 9:** Accuracy analysis for WGF-LN method with existing systems.

No of data from dataset	LegoNet	CNN	DNN	Proposed WGF-LN
100	88.532	92.946	90.165	95.329
200	89.853	93.529	91.543	96.087
300	90.176	94.965	92.243	96.432
400	91.721	95.032	93.094	97.533

### Comparative analysis for OPPORTUNITY dataset

In this section, the effectiveness of the proposed convolutional neural network, recurrent neural network, and long short-term memory based CNN (LSTM-CNN) is evaluated in comparison to the existing WGF-LN.

**Figure 10:**
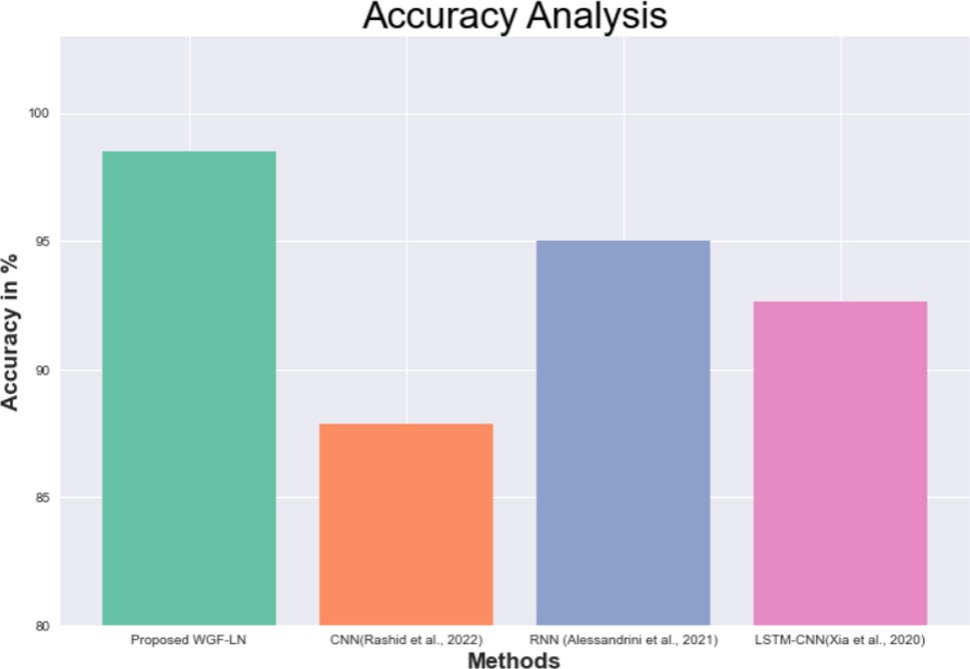
A comparative analysis of the proposed technique with the existing method.

Table 10 and Fig. 10 shows the performance analysis of the proposed technique with the existing methods using the same speech command dataset. In this analysis, the proposed WGF-LN shows greater than existed methods, with a 98.49% accuracy, and the accuracy of the existing method for the same data set using CNN is 87.86%. Likewise, the accuracy for the same dataset using different techniques namely RNN and LSTM-CNN is 95% and 92.63% respectively.

**Table 10:** A comparative analysis of the proposed technique with the existing method.

Methods	Accuracy (%)
Proposed WGF-LN	98.49
CNN(Rashid et al., 2022)	87.86
RNN (Alessandrini et al., 2021)	95
LSTM-CNN(Xia et al., 2020)	92.63

## Conclusion

In conclusion, the paper "Wasserstein gradient flow legonet-based Edge Driven Intelligence for Wearable Devices in Human Activity Recognition" suggests a plausible development path for human activity recognition. To increase the legitimacy and effectiveness of this initiative, however, three significant obstacles must be overcome. Priority number one is a greater emphasis on clarity. The technical aspects of the Wasserstein gradient flow legonet method should be conveyed in a manner accessible to a broader audience, including those unfamiliar with the complexities of the field. This will make the research findings and contributions more understandable and applicable. Second, a more substantial commitment to transparency is required. Detailed details of the datasets, preprocessing processes, and model parameters used in the study should be supplied, allowing fellow researchers to duplicate and validate the results. Sharing code, data, and pre-trained models encourages an environment of collaborative progress and examination, increasing the overall reliability of the conclusions. Scientific rigor is essential for the legitimacy of any scientific activity. To determine the superiority of the suggested strategy, rigorous experimental design, including complete benchmarks and comparison with existing methodologies, should be performed. The relevance of the contribution is directly related to the magnitude of its impact on the state-of-the-art, which reinforces the study's credibility. Ethical considerations should be thoroughly studied and incorporated into the study framework. Discussions about potential biases, privacy problems, and the ethical implications of using the technology in real-world scenarios are essential. Addressing these challenges proactively protects against unintended repercussions and reflects a responsible and forward-thinking attitude to research.

This experiment demonstrates that human activity can be detected using a novel WGF-LN classifier. The tasks mentioned above—data cleansing, integration, transformation, feature extraction, feature reduction, post-processing, and classification—are completed using the suggested methodology. The experimental analysis comes last. The performance of the proposed WGF-LN was compared to that of well-known techniques, and the results were then analyzed and commented on. When the proposed model is used for classification, it achieves an excellent accuracy of 98.82%, and when used for feature extraction, it performs an impressive accuracy of 84%. Not only does the proposed model produce the best results across all metrics, but it also evaluates the performance of each upgraded component using a variety of metrics to determine which ones are most useful. Based on the findings of all metrics, the proposed model outperforms the currently used methods for activity recognition classification. There was a strong emphasis on extracting and analyzing data using Edge, but no security issues were addressed. In summation, by adding these components of clarity, transparency, scientific rigor, and ethical reflection, the study on Wasserstein gradient flow legonet-based edge-driven intelligence for wearable devices in human activity recognition can increase its credibility and influence. A complete and accessible description of the methodology, transparent research processes, solid experimental validation, and ethical conscience all contribute to the growth of knowledge and the positive transformation of the area. Therefore, in the future, the work can be developed focusing on security-based issues using cryptographic techniques.

### Methods

All methods were carried out in accordance with relevant guidelines and regulations.

## Data Availability

The data used to support the findings of this study are available from the corresponding author upon request.
